# A role for histone acetylation in regulating transcription elongation

**DOI:** 10.1080/21541264.2017.1394423

**Published:** 2017-12-08

**Authors:** Michael C. Church, Alastair B. Fleming

**Affiliations:** a Stowers Institute for Medical Research, 1000 E 50th Street, Kansas City, MO, United States; b Department of Microbiology, Moyne Institute, Trinity College Dublin, University of Dublin, Dublin, Ireland

**Keywords:** *S. cerevisiae*, SAGA, Swi-Snf, histone acetylation, transcription elongation

## Abstract

Recently, we reported that a major function of histone acetylation at the yeast *FLO1* gene was to regulate transcription elongation. Here, we discuss possible mechanisms by which histone acetylation might regulate RNA polymerase II processivity, and comment on the contribution to transcription of chromatin remodelling at gene coding regions and promoters.

## Introduction

DNA in eukaryotic cells exists as a nucleoprotein complex called chromatin. The fundamental subunit of chromatin is the nucleosome which comprises two copies each of the core histones, H3, H4, H2A and H2B, around which is wrapped ∼147 bp of DNA [1]. This structure is generally considered refractory to any process that needs access to the DNA, including DNA repair, replication and gene transcription. However, the chromatin fibre is highly dynamic and can be ‘remodelled’ to either expose or mask the underlying DNA as and when required [2]. Chromatin can be remodelled by either (i) the addition of post-translational modifications (PTMs) to the histone proteins, or (ii) the activity of ATP-dependent remodelling complexes such as the Swi-Snf complex [3]. Swi-Snf uses the power from ATP hydrolysis to alter nucleosome positioning by either sliding nucleosomes along the DNA, or promoting partial or complete removal of the histone proteins from the nucleosome [4]. Histone modifications, on the other hand, can influence the properties of the nucleosome to alter chromatin function, or they can act as recognition sites for the binding of non-histone proteins which can alter chromatin structure and function [5]. Indeed, protein subunits of ATP-dependent remodelling complexes can contain domains which display an affinity for chromatin marked by the various histone PTMs thereby targeting chromatin remodelling activities to discrete sites within the genome [6].

The histone modifications most commonly associated with active gene transcription are histone acetylation and histone methylation [7]. Numerous enzymes have been identified that can catalyse the addition and removal of most of these modifications. Thus, the yeast genome is packaged via nucleosomes that are decorated with highly dynamic histone modifications that can regulate chromatin structure and function.

Arguably the most commonly studied aspect of chromatin remodelling has been the analysis of chromatin at gene promoters in response to gene activation and repression [8]. The general consensus of opinion was that promoter chromatin structure can regulate transcription by either occluding or allowing access of transcription factors to important DNA elements within the promoter to inhibit or enable transcription initiation, respectively. However, it has also been shown that during transcription elongation nucleosomes are evicted to allow passage of RNA polymerase II (Pol II), and are reassembled in its wake to maintain chromatin structure and prevent aberrant transcription initiation from occurring within transcribed genes [7]. Thus, the nucleosome dynamics required to ensure accurate gene transcription are regulated via a complex interplay between histone PTMs and non-histone proteins at both promoters and coding regions. However, the precise mechanism of action of the plethora of PTMs and chromatin remodellers associated with promoters or coding regions, and their relative contribution to transcription, remains largely unknown.

## Chromatin remodelling at the *FLO1* gene in *Saccharomyces cerevisiae*

We recently investigated the role of histone acetylation in the regulation of transcription of the yeast *FLO1* gene [9]. *FLO1* encodes a lectin-like cell wall protein which mediates a cell aggregation stress-response known as flocculation, whereby yeast cells within the ‘floc’ are protected against the outside environment [10]. This phenotype, although attenuated in laboratory strains, is important for industries such as brewing, and is of relevance to biofilm formation.

**Figure 1.**
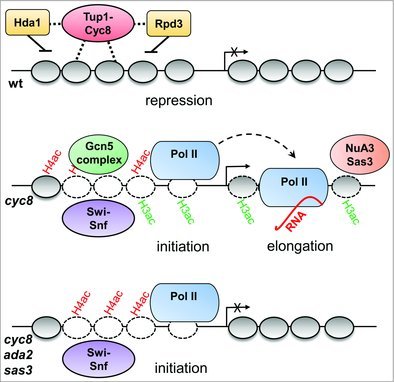
**Histone acetylation in the *FLO1* ORF is required for transcription elongation.** In wild-type (wt) cells, *FLO1* transcription is repressed via the action of the Tup1-Cyc8(Ssn6) co-repressor which cooperates with the histone deacetylases (HDACs), Hda1 and Rpd3, to establish an ordered array of hypoacetylated nucleosomes across the gene promoter. Loss of Tup1-Cyc8 (*cyc8*) leads to the enrichment of Gcn5- and Sas3-containing complexes located predominantly at the *FLO1* promoter and ORF respectively, concomitant with hyperacetylation of promoter and ORF nucleosomes. The ATP-dependent Swi-Snf chromatin remodelling complex is also recruited to the *FLO1* promoter, where it displaces/evicts promoter nucleosomes (white ovals). This strain exhibits robust Pol II occupancy at the *FLO1* promoter and ORF, and *FLO1* is transcribed. In a cell that lacks Cyc8, Gcn5-containing complexes, and Sas3 (*cyc8 ada2 sas3*), promoter nucleosomes are acetylated, and nucleosome loss at the *FLO1* promoter occurs as in a *cyc8* strain, whilst Pol II is detected in the promoter, but not in the ORF. Together, these data suggest Gcn5 and Sas3-dependent ORF histone acetylation are required to enable entry of Pol II into the *FLO1* ORF. The transcription start site is depicted as a black arrow with or without a cross to denote inactive or active transcription, respectively. Figure is adapted from Church *et al*., 2017 [9].

We had previously shown that *FLO1* transcription is regulated via the antagonistic action of the Tup1-Cyc8(Ssn6) co-repressor and Swi-Snf co-activator complexes which function to repress or activate transcription, respectively [11,12] (Figure 1). The repressed *FLO1* gene is associated with a Tup1-Cyc8 dependent array of strongly positioned, hypoacetylated nucleosomes which span the promoter and upstream region. Conversely, *FLO1* de-repression correlates with increased promoter histone acetylation, and the Swi-Snf dependent disruption of this nucleosome array. In a mutant deficient for Swi-Snf activity, nucleosome remodelling and *FLO1* transcription are abolished suggesting transcription of this gene is Swi-Snf dependent [11].

Based on the hypothesis that histone acetylation was positively required for *FLO1* transcription, we aimed to identify (i) which histone acetyltransferase (HAT) enzyme was required for transcription, (ii) which histone lysine (K) residues were involved, and (iii) to determine the mechanism of action of histone acetylation in *FLO1* transcription. We hypothesised that histone acetylation would drive Swi-Snf recruitment and nucleosome remodelling at the *FLO1* promoter, which would enable the initiation of transcription. Surprisingly, this proved not to be the case. Instead, our results suggested the dominant role for histone acetylation at *FLO1* was in the open reading frame (ORF) to enable Pol II elongation (Figure 1).

Specifically, we confirmed *FLO1* transcription was dependent upon the activities of the Gcn5 and Sas3 HATs. We found Gcn5 was predominantly enriched at the *FLO1* promoter, but was also detectable at low levels in the ORF. Sas3 on the other hand, was only detectable in the gene coding region. We also showed that histone H3 lysine 14 (H3K14) was the critical target for acetylation by these HATs that was required for *FLO1* transcription.

To investigate the role of these HATs in regulating transcription, we used the anchor-away technique to conditionally and rapidly deplete the Tup1 repressor from the nucleus so as to monitor the time-course of transcription-coupled chromatin events at the *FLO1* gene following de-repression. We then examined H3 acetylation, Swi-Snf recruitment, histone depletion, and Pol II occupancy at the promoter and ORF following gene activation in the presence and absence of Gcn5 and Sas3-dependent histone H3 acetylation.

Our data showed that following removal of the co-repressor from the promoter, there was a rapid acetylation and depletion of H3 which was accompanied by Swi-Snf recruitment to the promoter and Pol II enrichment in the promoter and ORF [9]. This correlated with increased *FLO1* transcription and expression of the flocculation phenotype. When the experiment was repeated in a strain deficient for both Gcn5 and Sas3 activities, although H3K14 acetylation (H3K14ac) and *FLO1* transcription were abolished, Swi-Snf recruitment, histone eviction and Pol II occupancy at the promoter persisted. However, we could not detect Pol II in the ORF. Together, this suggested that Swi-Snf recruitment was not dependent on Gcn5 and Sas3-dependent histone H3K14ac, and that histone eviction at the promoter was insufficient to enable *FLO1* transcription. Instead, these data were consistent with histone H3K14ac in the ORF being a critical requirement for transcription elongation.

## How could histone acetylation regulate entry of Pol II into the ORF?

The absence of Pol II in the *FLO1* ORF following the removal of the Tup1-Cyc8 co-repressor when Sas3 and Gcn5 dependent H3K14ac was abolished could be due to either (i) a failure to release Pol II from the promoter, or (ii) prevention of Pol II from entering into the ORF. We favour the latter possibility based on evidence of a role for histone acetylation in the eviction of ORF nucleosomes during transcription elongation. Indeed, it has been shown that NuA3 occupancy can be detected globally at gene coding regions [13]. Furthermore, there is evidence for Gcn5, possibly in the context of the SAGA complex, being required for histone acetylation within inducible gene ORFs where it promotes histone eviction. For example, in the absence of Gcn5, acetylation-dependent histone eviction at the *GAL1* ORF is decreased, and the Pol II occupancy profile across the ORF is altered, indicating Pol II fails to fully traverse the coding region [14]. Studies in *S. pombe* have also shown that Gcn5 is required for ORF nucleosome eviction and Pol II progression at stress responsive genes [15]. A similar role has also been shown for the NuA4 complex, which is responsible for H4 acetylation [16]. This complex was found at gene coding regions where it was shown to play a role in histone eviction during transcription elongation. Importantly, strains deficient for Gcn5 and Esa1, which is the catalytic subunit of NuA4, show a 6-AU sensitivity, which is indicative of defective transcription elongation. In addition, an *esa1* mutant shows defects in Pol II processivity consistent with a failure of Pol II reach the end of the active genes tested. Most strikingly, Pol II has a slower elongation rate in the *esa1* mutant [16]. Presumably the above defects in Pol II processivity are all a consequence of the failure to adequately remove the nucleosomes blocking the path of elongating Pol II.

We therefore hypothesise that a major role of Sas3 and Gcn5 is to acetylate H3K14 within the *FLO1* gene coding region which is required to drive histone eviction to allow Pol II progression across the ORF. If this hypothesis is correct, we would predict that following *FLO1* activation we would see increased H3 occupancy in the *FLO1* ORF in an H3K14ac deficient mutant. It would also be pertinent to confirm in the histone acetylation-deficient background if the Pol II at the promoter is initiation competent, and to determine if Pol II enters the ORF, and if so, to measure how far it travels.

## In what context do Gcn5 and Sas3 act upon the *FLO1* gene?

The Sas3 protein is the catalytic subunit of the yeast NuA3 histone acetyltransferase complex which predominantly acetylates histone H3 K14 and K23 [17]. Gcn5 is the HAT activity-containing subunit of the evolutionary conserved SAGA complex, and related ADA and SLIK complexes, which can acetylate histones H3 and H2B [18,19]. Two functionally distinct NuA3 complexes have been identified, NuA3a and b, which contain domains that have affinities for histone modifications found at gene promoters and ORFs respectively [20]. The SAGA complex has been mapped to the upstream activating sequences (UASs) of most Pol II transcribed genes, and has been proposed to act as a general cofactor for all Pol II transcription [21,22]. Importantly, SAGA occupancy does not necessarily correlate with Gcn5 activity, suggesting that Gcn5 can either act at a distance from where it is located, or is resistant to detection at some regions due to its highly dynamic interaction with the chromatin fibre [23,24]. Thus, we would predict Sas3 is functioning at the *FLO1* ORF in the context of the NuA3b complex, whereas Gcn5 is functioning in the context of the SAGA complex acting from its primary location in the promoter to acetylate histones in the 5’ ORF. SAGA(Gcn5) might also be present in the ORF at greater levels than our chromatin immunoprecipitation (ChIP) analysis suggests, perhaps due to it being difficult to detect in this region using this technique.

## How are the HATs targeted to the active *FLO1* gene?

It has been proposed that promiscuous HAT and histone deacetylase (HDAC) activity across the genome maintains a steady state level of global chromatin acetylation [25]. Localised increases in histone acetylation could arise from the specific recruitment of HATs, or removal of HDACs, at target areas such as the promoter and ORFs of active genes. The occupancy we see of Gcn5 predominantly at the active *FLO1* promoter is consistent with the occupancy of Gcn5 seen at almost all active Pol II-dependent genes where SAGA can be recruited by transcription factors to upstream activator sequences [21,26]. However, the identity for the recruiting factors of Gcn5 at *FLO1* have not been identified.

HAT occupancy within ORFs could result from tethering of the HATs to Pol II at the promoter, which then enter into the coding region. Alternatively, HATs could be recruited directly to actively transcribed gene coding regions via chromatin which has been post-translationally modified in some way. The latter mechanism may be more applicable to both the low occupancy we see of Gcn5 in the active *FLO1* ORF, and the high occupancy we see of Sas3. Indeed, the observed occupancy of Gcn5 at the 5’ ORF is consistent with its recruitment by the Sgf29 sub unit of SAGA which binds to H3K4 methylated (me) chromatin which we would expect to be enriched in this region of the gene when active [27].

With regards Sas3 occupancy, the Yng1 and Taf14 subunits common to NuA3 complexes contain a PhD finger, and YEATS domain respectively, whilst the unique Pdp3 sub-unit of NuA3b contains a PWWP domain. *In vitro* evidence shows that the YEATS domain of Taf14 can bind to acetylated H3K9, whereas *in vitro* and *in vivo* evidence shows that the Phd finger of Yng1 and the PWWP domain of Pdp3 can respectively target Sas3 to H3K4 and H3K36 methylated chromatin within gene coding regions [13]. Interestingly, mapping of the global occupancy profile of NuA3 subunits has found this complex was most enriched in the middle of gene coding regions of highly active, long genes [13]. Considering the *FLO1* gene is 4.1 kb long and is marked by H3K9ac and H3K36me when active (Fleming, unpublished data), it would fulfil the criteria for a Sas3-enriched gene. Thus, we would propose that at *FLO1*, Gcn5 and Sas3 might be recruited to the actively transcribed gene coding region by the transcription-coupled deposition of H3K4me and H3K36me expected to be found at the 5’ and 3’ ORF regions. Histone acetylation in the 5’ ORF might also be a consequence of Gcn5 acting at a distance from its location in the promoter.

## How could histone acetylation promote histone eviction?

It has been proposed that histone acetylation contributes to the recruitment of ATP-dependent chromatin remodelling complexes which bring about nucleosome repositioning, disassembly or eviction. For example, the Snf2 subunit of the Swi-Snf complex contains a bromodomain which has an affinity for H3 acetylated chromatin which has been shown to determine its association with active gene promoters and DNA double-strand break sites [28,29]. The RSC ATP-dependent chromatin remodelling complex also contains bromodomains in the Sth1, Rsc1, Rsc2 and Rsc4 subunits, and is localised in an acetylation-dependent manner to gene coding sequences where it regulates histone and Pol II occupancy [16,30]. Interestingly, the tandem bromodomains within the RSC4 subunit have been biochemically shown to preferentially bind H4K14ac [31]. Furthermore, the central ATPase of the human Swi-Snf (BAF) chromatin remodelling complex contains a bromodomain that also shows specificity for H3K14ac, highlighting the potential importance of this mark upon ATP-dependent chromatin remodelling complex recruitment [32].

**Figure 2.**
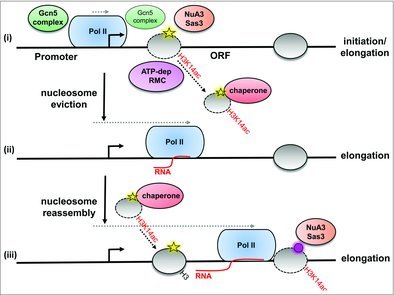
**Model to show the possible mechanism of action of histone acetylation in regulating transcription elongation.** (i) Early elongating Pol II meets a nucleosomal barrier in the ORF, and histone H3 is acetylated at lysine 14 (H3K14ac) by the activities of the HATs, Gcn5 and Sas3, in the context of the SAGA and NuA3 complexes. ORF nucleosomes containing H3K4me (yellow star) may aid in the recruitment of NuA3 and SAGA to the 5’ ORF. H3K14ac promotes recruitment of ATP-dependent chromatin remodelling complexes (ATP-dep RMC) to the ORF to drive nucleosome eviction. (ii) The evicted histones are tethered to histone chaperones and Pol II advances along the ORF. (iii) The histones are reassembled in the wake of elongating Pol II, and the resultant nucleosomes are deacetylated [39]. The process is repeated when Pol II reaches the next nucleosome, which has been depicted to contain H3K36me (purple circle) to illustrate possible events at the 3’ ORF, where H3K36me may aid in recruitment of NuA3. The transcription start site is denoted as a black hooked arrow. Refer to main text for details.

The *in vivo* nucleosome eviction activity of such chromatin remodelling complexes has been shown to require histone chaperones to tether the evicted histones [7]. One of the best characterised histone chaperones is Asf1, which coordinates nucleosome dynamics at gene promoters and open reading frames [33,34]. Indeed, Swi-Snf has been shown to cooperate with, or work in parallel to, Asf1 to displace nucleosomes at the *HO* promoter and *PHO5*/*PHO8* promoters, respectively [35,36]. More recent work in fission yeast has also provided direct *in vivo* evidence to show that the Fun30^Fft3^ ATP-dependent chromatin remodelling complex cooperates with the FACT histone chaperone to promote nucleosome eviction within ORFs to enable Pol II elongation [37]. Thus, we hypothesise that the concerted action of histone H3K14ac, ATP-dependent chromatin remodelling complexes and histone chaperones would be required to promote histone eviction at the ORF to allow Pol II elongation (Figure 2). However, aside from Swi-Snf having been shown to be present and active at the *FLO1* promoter, which ATP-dependent remodellers or histone chaperones are functional at the *FLO1* ORF is currently unknown.

## Is there a pioneering round of transcription elongation?

We have proposed a model in which Sas3, and to a lesser extent Gcn5, are recruited to the active *FLO1* gene coding region via the deposition of transcription-coupled histone methylation marks (Figure 2). We further propose the resultant acetylation of nucleosomes in the ORF is required to drive histone eviction to enable Pol II passage across the gene. However, this model raises the fundamental question of how eviction would occur ahead of the first polymerase to cross an ORF upon gene activation, which would presumably be in the absence of the pre-requisite transcription-coupled methylation marks. To account for this, it could be that a ‘pioneering’ round of Pol II elongation occurs in which either (i) a unique Pol II, or Pol II associated with unique auxiliary proteins, can bring about eviction in the absence of increased acetylation or (ii), the HATs are already tethered to the ‘pioneering’ Pol II. Alternatively, the PTMs required for passage of the first polymerase are established following prior transcription shutdown. This deposited PTM signature might then poise the gene for rapid transcription in the future. Intriguingly, the Tup1-Cyc8 complex has been shown to prime the repressed cell-type specific genes which are under its negative control, via Gcn5-dependent pre-acetylation of histones at the repressed gene promoters [38]. Thus, the repressor of *FLO1* transcription may also be required for gene activation.

## Conclusion

Our data indicate that Sas3 and Gcn5-dependent H3K14ac in the gene coding region is required to enable transcription elongation of the Swi-Snf dependent *FLO1* gene. We have shown that in a strain deficient for Sas3 and Gcn5 activities, Pol II could not be detected in the ORF even though Swi-Snf recruitment, histone eviction and Pol II occupancy still occurred at the promoter. Thus, Swi-Snf recruitment and activity at the promoter does not require Sas3 and Gcn5-dependent H3K14ac, and *FLO1* transcription is not solely dependent upon promoter histone eviction. We propose that promoter and ORF chromatin remodelling are independent events which are critical for *FLO1* transcription initiation and elongation, respectively. A challenge for the future will be to determine the interplay between the histone PTMs, chromatin remodelling complexes and histone chaperones that correlate with transcription elongation, whilst taking into account the concomitant nucleosome eviction and reassembly taking place.
